# Simultaneous Ozone and High Light Treatments Reveal an Important Role for the Chloroplast in Co-ordination of Defense Signaling

**DOI:** 10.3389/fpls.2022.883002

**Published:** 2022-07-07

**Authors:** Enjun Xu, Mikko Tikkanen, Fatemeh Seyednasrollah, Saijaliisa Kangasjärvi, Mikael Brosché

**Affiliations:** ^1^Organismal and Evolutionary Biology Research Programme, Faculty of Biological and Environmental Sciences, Viikki Plant Science Centre, University of Helsinki, Helsinki, Finland; ^2^Department of Biochemistry, Molecular Plant Biology, University of Turku, Turku, Finland; ^3^Institute of Biotechnology, HILIFE – Helsinki Institute of Life Science, University of Helsinki, Helsinki, Finland

**Keywords:** ozone, cell death, signal interaction, high light, abscisic acid, Arabidopsis

## Abstract

Plants live in a world of changing environments, where they are continuously challenged by alternating biotic and abiotic stresses. To transfer information from the environment to appropriate protective responses, plants use many different signaling molecules and pathways. Reactive oxygen species (ROS) are critical signaling molecules in the regulation of plant stress responses, both inside and between cells. In natural environments, plants can experience multiple stresses simultaneously. Laboratory studies on stress interaction and crosstalk at regulation of gene expression, imply that plant responses to multiple stresses are distinctly different from single treatments. We analyzed the expression of selected marker genes and reassessed publicly available datasets to find signaling pathways regulated by ozone, which produces apoplastic ROS, and high light treatment, which produces chloroplastic ROS. Genes related to cell death regulation were differentially regulated by ozone versus high light. In a combined ozone + high light treatment, the light treatment enhanced ozone-induced cell death in leaves. The distinct responses from ozone versus high light treatments show that plants can activate stress signaling pathways in a highly precise manner.

## Introduction

To be a plant is to prioritize between conflicting obligations – developmental programs should be balanced with inputs from the environment including varying light conditions and unfavorable growth conditions such as extreme temperatures, lack of water or pathogen attack. Maintaining permanently active defenses diverts energy from growth and typically plants with constitutively activate defenses are dwarfed. Hence, activation of defenses should only take place when necessary. Multiple signaling molecules take part in plant responses to the environment, including reactive oxygen species (ROS), Ca^2+^, and plant hormones, such as abscisic acid (ABA), salicylic acid (SA), jasmonic acid (JA), and ethylene.

Reactive oxygen species including hydrogen peroxide, superoxide, and singlet oxygen (H_2_O_2_, O_2_.^–^, and ^1^O_2_, respectively), are continuously formed as metabolic by-products in mitochondria, chloroplasts, and peroxisomes. However, the emerging consensus is that ROS are also actively produced as signaling molecules in the apoplast and chloroplasts to drive developmental processes and plant responses to abiotic and biotic stress (Phua et al., 2021). Each type of ROS can be characterized by its own reactivity and lifetime (Waszczak et al., 2018). Additionally the site of ROS production, e.g., apoplast, chloroplast, mitochondria, or peroxisome, leads to activation of different signaling pathways (Gadjev et al., 2006; Vaahtera et al., 2014). The apoplastic ROS burst is an early response to recognition of many pathogens *via* pathogen-associated molecular patterns (PAMPs), and is produced by cell wall peroxidases and respiratory burst oxidase homologue D (RBOHD). The air pollutant ozone (O_3_) breaks down in the apoplast to O_2_.^–^ and H_2_O_2_, followed by activation of the cells own ROS production machinery (Wohlgemuth et al., 2002; Vainonen and Kangasjarvi, 2015). While ozone levels at heavily ozone polluted areas vary between 60 and 100 ppb (Tiwari et al., 2008), higher concentrations up to 350 ppb O_3_ have been used as a research tool to activate and study apoplastic ROS signaling (Vainonen and Kangasjarvi, 2015; Xu et al., 2015a). In chloroplasts, formation of ROS is commonly associated with high light (HL) stress. In addition, the chloroplast regulates many aspects of plant defense and pathogen responses. This includes the biosynthesis steps for several stress hormones, including ABA, JA, and SA (Littlejohn et al., 2021). In pathogen infections, not only RBOHD and cell wall peroxidases generates apoplastic ROS, but also the chloroplast provides ROS for defense signaling (Zabala et al., 2015; Littlejohn et al., 2021). The photosynthetic machinery is dynamically regulated in order to keep energy transduction reactions in balance and to minimize ROS-producing side reactions (Tikkanen and Aro, 2014). Nevertheless, the regulatory machinery not only functions against ROS production, but can also be an important factor controlling the initiation and strength of ROS signals from chloroplast (Frenkel et al., 2009; Tikkanen et al., 2014; Gollan et al., 2015). There is no scientific consensus for what constitutes a HL treatment to initiate chloroplast retrograde or ROS signaling, but what is often used to study HL stress in *Arabidopsis thaliana* is a shift from fairly low light conditions (50–100 μmol m^–2^ s^–1^) to HL (>1,000 μmol m^–2^ s^–1^) (Table 1). Under these conditions, the HL treatment leads to production of ^1^O_2_, O_2_.^–^, and H_2_O_2_ in the chloroplast and results in large scale changes in gene expression (Table 1; Waszczak et al., 2018). In addition, the HL treatment also activates systemic signaling that regulates several biological processes including expression of defense related genes (Karpinski et al., 1999; Zandalinas et al., 2019b).

**TABLE 1:** Growth conditions used for Arabidopsis HL experiments and transcriptome analysis with qPCR, microarrays or RNA-seq.

References	Soil/*in vitro*	Age	Light period	Light level (during growth)	Light level (stress treatment)	Time point	Additional information
This study (Helsinki-1)	Soil	3 weeks	12 h light/12 h dark	230 μmol m^–2^ s^–1^	230 μmol m^–2^ s^–1^ (O_3_, 350 nL L^–1^)	1 h	This experiment tested the effect of O_3_ only
This study (Helsinki-2)	Soil	3 weeks	12 h light/12 h dark	110 μmol m^–2^ s^–1^	1,100 μmol m^–2^ s^–1^ (O_3_, 350 nL L^–1^)	1 h	HL treatment + 2°C compared to control
This study (Helsinki-3)	Soil	3 weeks	12 h light/12 h dark	110 μmol m^–2^ s^–1^	1,100 μmol m^–2^ s^–1^ (O_3_, 350 nL L^–1^)	1 h	HL treatment + 10°C compared to control
This study (Turku)	Soil	4 weeks	8 h light/16 h dark	130 μmol m^–2^ s^–1^	1,300 μmol m^–2^ s^–1^	1 h	HL treatment + 3°C compared to control
Bechtold et al., 2008	Soil	5–6 weeks	8 h light/16 h dark	150 μmol m^–2^ s^–1^	750 or 2,000 μmol m^–2^ s^–1^	45 min	
***Microarray experiments***							
Kleine et al., 2007	*In vitro*	7 days	Constant light	100 μmol m^–2^ s^–1^	1,000 μmol m^–2^ s^–1^	3 h	
Van Aken et al., 2013	Soil	3 weeks	16 h light/8 h dark	100 μmol m^–2^ s^–1^	1,000 μmol m^–2^ s^–1^	1 h	
Tikkanen et al., 2014	Soil	6 weeks	8 h light/16 h dark	130 μmol m^–2^ s^–1^	1,000 μmol m^–2^ s^–1^	1 h	
***RNA-seq experiments (letters in brackets refer to Figure 3*)**							
Zandalinas et al., 2019a (A)	Peat pellets	4–5 weeks	8 h light/16 h dark	50 μmol m^–2^ s^–1^	2,000 μmol m^–2^ s^–1^	2, 4, and 8 min	
Zandalinas et al., 2020 (B)	Peat pellets	40–55 days	16 h light/8 h dark	50 μmol m^–2^ s^–1^	1,700 μmol m^–2^ s^–1^	2 and 8 min	
Fichman et al., 2020 (C)	Peat pellets	4 weeks	10 h light/14 h dark	50 μmol m^–2^ s^–1^	1,700 μmol m^–2^ s^–1^	2, 8, and 30 min	
Crisp et al., 2017 (D)	Soil	3 weeks	12 h light/12 h dark	100 ± 25 μmol m^–2^ s^–1^	1,000 μmol m^–2^ s^–1^	30 min to 2 h + several recovery time points	
Huang et al., 2019 (E)	*In vitro*	7 days	Constant light	60 μmol m^–2^ s^–1^	1,200 μmol m^–2^ s^–1^	30 min to 72 h	Temperature in HL treatment maintained at control
Crisp et al., 2017 (F)	Soil	3 weeks	12 h light/12 h dark	100 ± 25 μmol m^–2^ s^–1^	1,000 μmol m^–2^ s^–1^	1 h	
Alvarez-Fernandez et al., 2021 (G)	Soil	35 days	8 h light/16 h dark	150 μmol m^–2^ s^–1^	1,100 μmol m^–2^ s^–1^	3.5 h	HL treatment + 5°C compared to control
Zandalinas et al., 2021b (H)	*In vitro*	9–10 days	Information not provided*	50 μmol m^–2^ s^–1^	700 μmol m^–2^ s^–1^	1.5 h	
Balfagón et al., 2019 (I)	Peat pellets	30 days	12 h light/12 h dark	50 μmol m^–2^ s^–1^	600 μmol m^–2^ s^–1^	7 h	HL treatment + 4°C compared to control

*For comparison, the growth conditions used for qPCR after HL or O_3_ treatments in Figures 2, 4, 5, 8 is also provided. Potential increase in temperature during HL treatment is included as additional information for the experiments where this information was available.*

*Information on light period not provided, but the authors refer to a previous publication where constant light was used.

There is an ongoing discussion in the literature to which extent there is interaction between HL and heat stress signaling. From an environment point of view, the interaction is clear, as days with very HL levels would typically also be warmer (Zandalinas et al., 2021a). In laboratory experiments, there is a similar question about the interaction as depending on the light source, in HL experiments there can also be an associated increase in temperature unless precaution is taken to control the temperature or filter the light through water (see for example, Jung et al., 2013). At the molecular level, using marker genes to report transcriptional changes, there is interaction between HL and heat stress, as the commonly used marker gene *APX2* (*ASCORBATE PEROXIDASE 2*) shows synergistic interaction with higher transcript levels when HL is combined with increased temperature (Jung et al., 2013; Huang et al., 2019). From array and RNA-seq experiments in different combined stresses including HL + heat stress, there is an emerging consensus that stress combination leads to a transcriptional response that is distinct from single stress treatments and cannot be predicted from experiments using only single treatments (reviewed in Balfagon et al., 2020; Zandalinas et al., 2021b). Regulators of the interaction between several stresses is suggested to include ABA, JA, and ROS (Balfagon et al., 2020). In this context, combined treatments with O_3_ and HL makes it possible to test the interaction of ROS from two distinct subcellular locations, as O_3_ will lead to ROS signals from the apoplast and HL from the chloroplast. The O_3_ and HL interaction could also be of ecological relevance, as days with more light are associated with increased O_3_ levels (Tiwari et al., 2008).

The availability of Arabidopsis mutants has significantly facilitated the discovery of proteins that act in specific ROS signaling pathways. The EXECUTER proteins act in the chloroplast to mediate ^1^O_2_ signaling (Zhang et al., 2014). After HL treatment, in the nucleus Topoisomerase VI acts as a positive regulator of ^1^O_2_ responsive genes but as negative regulator of H_2_O_2_ regulated genes (Simkova et al., 2012). Similarly, the small zinc finger proteins METHYLENE BLUE SENSITIVITY 1 and 2, regulate ^1^O_2_ but not H_2_O_2_ responsive genes after HL treatment (Shao et al., 2013). Studies have also revealed key signaling roles for chloroplast-derived metabolites, including triosephosphates, 3′-phosphoadenosine 5′-phosphate (PAP), β-cyclocitral, and methylerythritol cyclodiphosphate (MEcPP) (Leister, 2019). RCD1 (RADICAL-INDUCED CELL DEATH1), a protein that interacts with multiple transcription factors, also forms a regulatory node in signaling and differentially regulates plant responses to ROS from different subcellular sources (Shapiguzov et al., 2019). Several transcription factors regulate various aspects of the HL transcriptional response: heat shock transcription factors regulate the early response (Jung et al., 2013), BBX32 regulate down-regulation of genes associated with defense to pathogens (Alvarez-Fernandez et al., 2021), and HY5 associated with blue light and UV-B receptors (Kleine et al., 2007). A large collection of mutants defective in hormone biosynthesis, perception or down-stream signaling are also available, and for example, ABA biosynthesis or signaling mutants are impaired in expression of HL responsive genes and show increased damage by HL treatment (Galvez-Valdivieso et al., 2009; Huang et al., 2019).

Even though recent discoveries have identified a number of regulators in ROS signaling, there are still large gaps in our understanding how plants perceive and signal the presence of ROS as triggered by different types of external factors, and how plants prioritize between potentially conflicting defense signals. Here we applied O_3_ and HL treatments together with analysis of marker gene expression and transcript profiles to study the signaling effects elicited by apoplastic versus chloroplastic ROS. We also studied the effect of combined O_3_ + HL treatments to dissect possible interactions between these treatments. We further performed the combined treatments at different temperatures, as the HL response is strongly influenced by temperature. We show that genes related to cell death regulation were differentially regulated by O_3_ versus HL, that HL enhanced cell death caused by O_3_, and HL repressed the effect of O_3_ on transcriptional regulation of pathogen and cell death related genes.

## Materials and Methods

### Plant Growth

Seeds of *A. thaliana* Col-0 and mutants were obtained from Nottingham Arabidopsis Stock Centre or were a gift from Prof. Hannes Kollist *pyr pyl 112458* (Gonzalez-Guzman et al., 2012). The *coi1-16* and *slac1-3* mutants were previously described (Vahisalu et al., 2008; Xu et al., 2015a). In Helsinki, seeds were sown on 1:1 peat: vermiculite, stratified for 3 days, and then grown at 22/19°C (day/night), relative humidity of 70/90% (day/night), under a 12-h light/12-h dark cycle for a week. Subsequently the geminated seedlings were transplanted into new 1:1 peat: vermiculite mixture. All plants were grown in controlled chamber (Weiss Bio1300; Weiss Gallenkamp), at 22/19°C, and relative humidity of 70/90%, under a 12-h light/12-h dark cycle. Light levels in different conditions are outlined in Table 1; Helsinki-1, used 230 μmol of photons m^–2^ s^–1^, Helsinki 2 and 3 used 110 μmol of photons m^––2^ s^––1^. For O_3_ and combined O_3_ + HL experiments, treatments with 1 h 350 nL L^––1^ O_3_ were performed with 3-week-old plants. LED white lights^1^ were placed inside Weiss chambers to provide the HL treatment (1,100 μmol of photons m^–2^ s^–1^). The LED lights also increased the temperature at plant level with +10°C (growth condition Helsinki-3), to minimize the influence of temperature experiments were also performed with cooling of the LED lights (growth condition Helsinki-2). For quantification of cell death plants were treated 2 h 300 nL L^–1^ O_3_, 1,100 μmol of photons m^–2^ s^–1^ or both simultaneously. Subsequently plants were put to 50 ml tubes with 15 ml MilliQ-water and ion leakage was measured 6 h after the start of the treatments with a conductivity meter (Mettler Toledo LE703). In Turku (growth condition Turku, Table 1), plants were grown under 130 μmol of photons m^–2^ s^–1^ at 20°C, 8/16 h light/dark cycle, and 50% humidity. Four-week-old plants were shifted to a light intensity of 1,300 μmol of photons m^–2^ s^–1^ at 20°C for 1 h, this was associated with an increase in temperature of 3°C.

### RNA Isolation and qPCR

Gene expression analysis of selected marker genes was performed with qRT-PCR (Supplementary Table 1 includes primer sequences and primer amplification efficiencies). RNA was isolated with GeneJET Plant RNA Purification Mini Kit (ThermoFisher Scientific). RNA (2 μg) was DNAseI treated and reverse transcribed with Maxima Reverse Transcriptase and Ribolock Rnase inhibitor (ThermoFisher Scientific) and the reaction diluted to the final volume of 100 μl. qPCR was performed in triplicate using 5x HOT FIREPol EvaGreen qPCR Mix Plus (Solis Biodyne). The cycle conditions with Bio-Rad CFX384 were: 1 cycle initiating with 95°C 10 min, 45 cycles with 95°C 15 s, 60°C 30 s, 72°C 30 s and ending with melting curve analysis. Normalization of the data was performed in qBase 2.3 (Biogazelle^2^), with the reference genes *TIP41*, *YLS8*, and *SAND* for the O_3_ experiment and *PP2AA3*, *TIP41*, and *YLS8* in the HL and combined O_3_ + HL experiments. Primer amplification efficiencies were determined in qBase from a cDNA dilution series. qBase provides the qPCR results as calibrated normalized relative quantity for each gene (Hellemans et al., 2007). To facilitate comparison between treatments, fold induction by treatment was calculated as treatment/control. Statistical analysis of the qPCR was performed on log2 transformed data with *t*-test, one-way or two-way ANOVA in GraphPad Prism 6.07.

### Transcriptome and Cluster Analysis

Raw data from the Affymetrix ATH1-121501 and Agilent Arabidopsis 4 × 44K chips platform was obtained from several data sources. NASCARRAYS-392 (BTH treatment).^3^ From Gene Expression Omnibus: GSE39385 (SA 3 h); GSE19520 (ABA 3 h); GSE28800 (ABA 6 h); GSE45543 (ABA 6h); GSE5684 (Botrytis cinerea infection); GSE7743 (*cry1* and *hy5* treated with HL for 6h); GSE14247 (Ethylene 4 h); GSE5615 (Flg22); GSE19109 (*lht1*); GSE10646 (*mkk1 mkk2*); GSE32566 (Na_2_S); GSE18978 (*Pseudomonas syringae* ES4326); GSE14961 (SA 24 h); GSE6583 (*siz1*); GSE46107 (*wrky40* and *wrky63*).^4^ From ArrayExpress: E-ATMX-13 (MeJA).^5^ Raw data for *acd11* (Palma et al., 2010) was obtained from John Mundy. Raw data for *stn7*, *npq4*, and *tap38* treated with HL for 1 h (Tikkanen et al., 2014). RNA-seq raw data from O_3_ treatment for 2 h was obtained from Gene Expression Omnibus: GSE61542 (Col-0, C24, and Te) and GSE65740 (Col-0, *coi1-16 ein2 sid2*, and *tga2 tga5 tga6*) (Xu et al., 2015a,b). The pre-processing of Affymetrix data was performed with “robust multiarray average” normalization using affy package in R (Gautier et al., 2004). The Agilent microarray data was processed using the “half” background correction method and followed by “quantile normalization” using Limma package in R. RNA-seq data analysis of O_3_ data was performed with several packages in the JAVA-based client-server system Chipster (Kallio et al., 2011) and as previously described (Xu et al., 2015a). Differential expression for each experiment was computed by log2-base fold changes in a linear model between treatment and control, or between wild type and mutants. The false discovery rate of differentially expressed genes for treatment/control and between-treatment comparisons was based on the Benjamini and Hochberg (BH) method. The genes in Col-0 with a B-score >0 in response to O_3_ (GSE61542) and HL treatments [GSE46107 and (Tikkanen et al., 2014)] were selected as significantly expressed genes. The shared set of genes with oppositely regulated expression between O_3_ and HL treatments were extracted as candidate genes for comparing stress responses induced by O_3_ and HL. The processed data was discretized and clustered using Bayesian agglomerative hierarchical clustering algorithm (Wrzaczek et al., 2010). Gene Ontology (GO) term enrichment was performed using the AgriGO website with advanced settings of “Plant GO slim” in GO type (Du et al., 2010).

High light or excessive light RNA-seq data were downloaded as raw fastq files from GEO using accession IDs (Supplementary Table 2). The quality of fastq files was assessed using FastQC tool version v0.11.8. Raw reads were aligned to Arabidopsis reference genome (TAIR10.51) using STAR aligner version 2.7.8a and the quality of BAM files was checked using RseQC tool. Gene-level expression abundances were estimated using HTSeq tool, union mode (Putri et al., 2022). For identification of differentially expressed genes, raw read counts were imported to R/Bioconductor package Limma version 3.48.3 (Ritchie et al., 2015). Genes with insufficient number of read counts were filtered out before running the statistical test. This was done using filterByExpr() function from edgeR package with default parameters following the Limma package manual (Robinson et al., 2010; Ritchie et al., 2015). Differentially expressed genes with Benjamini-Hochberg FDR < 0.05 and log2 fold change > ±1 were extracted and used to find overlap between different datasets using InteractiVenn (Heberle et al., 2015).

## Results

### Expression of Light, Reactive Oxygen Species, Ozone, Heat Shock, and Pathogen Responsive Marker Genes in Abiotic, Biotic, and Hormone Treatments

**FIGURE 1:**
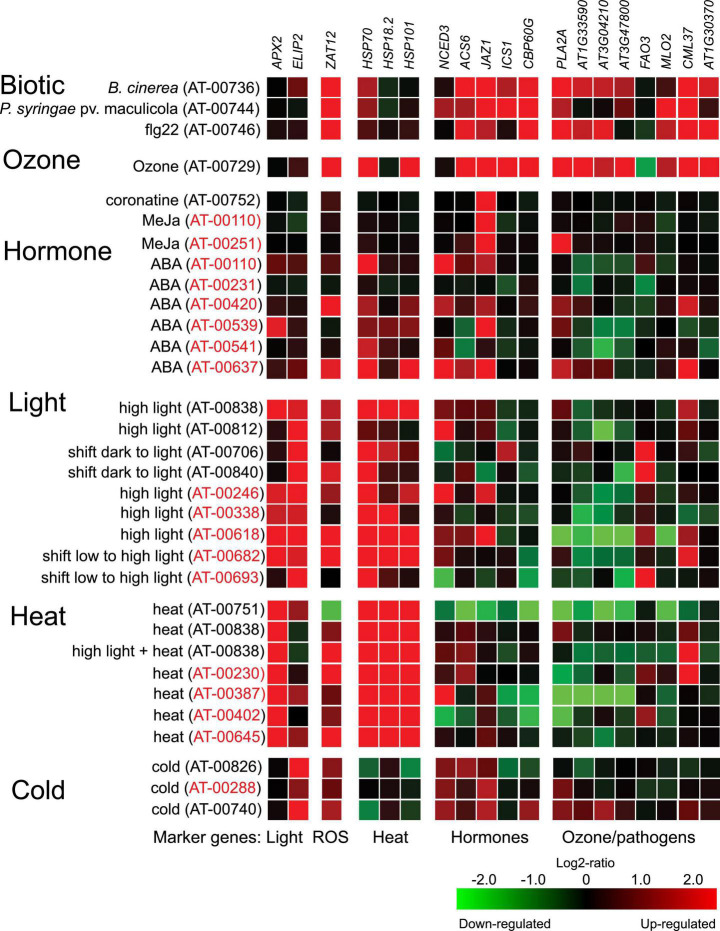
Expression of marker genes in publicly available transcriptome data. Experiments from the Genevestigator perturbation tool were selected to include O_3_, HL, heat, cold, hormones, and biotic stress treatments. The identification number for each experiment refers to the identifier in the Genevestigator database. Samples with identifiers in red comes from Affymetrix ATH1 array and in black from RNA-seq experiments.

A current limitation in our understanding of plant stress responses is the heterogeneity of experimental conditions used to grow plants for experiments. We collected information on experimental protocols for Arabidopsis HL experiments in Table 1, which included a variety of different growth conditions and day lengths. As intracellular ROS levels are higher in short day grown plants and cell death is regulated differently in short versus long day grown plants (Michelet and Krieger-Liszkay, 2012; Krasensky-Wrzaczek and Kangasjarvi, 2018), it is likely that growth conditions used to generate plants for experiments will influence the responses to subsequent HL treatment. To probe the molecular responses to light/photooxidative stress (initiated from chloroplasts) versus O_3_ treatments (that initiate ROS signaling from the apoplast), we selected several marker genes used in previous publications related to light and O_3_ treatments (Xu et al., 2015a), and visualized their expression levels in response to abiotic, biotic, and hormone treatments using data from the Genevestigator database (Figure 1; Hruz et al., 2008). As expected, marker genes used for HL stress (*APX2*, *ELIP2*, and *ZAT12*) were regulated in a majority of HL experiments, although with some exceptions. For example, *APX2* was not regulated in Genevestigator accession AT-00812 which contain the data from Huang et al. (2019). Transcript levels for *APX2* were also regulated by heat, consistent with regulation of *APX2* by heat shock transcription factors (Jung et al., 2013). Transcript levels for *ELIP2* was also regulated by cold, and this marker gene is proposed to have increased expression by several environmental stresses related to photoinhibition (Hayami et al., 2015). *ZAT12* transcript levels were regulated by all abiotic and biotic treatments, accordingly *ZAT12* is often referred to as a general ROS marker gene (Lim et al., 2019). To investigate the regulatory context of *APX2*, *ELIP2*, and *ZAT12* we used the updated version of the Arabidopsis Coexpression Tool (Zogopoulos et al., 2021), which identifies genes that are co-expressed with the target gene (Supplementary Figure 1). Consistent with the regulatory context from Genevestigator (Figure 1), *APX2* was co-regulated with numerous heat shock proteins (Supplementary Figure 1); *ELIP2* was co-regulated with many flavonoid biosynthesis genes that lead to production of pigments that protect against light and oxidative stress (Ferreyra et al., 2021); and *ZAT12* with genes related to biotic stress and hormone responses. Collectively, this suggests that using these three marker genes will report on different parts of the Arabidopsis transcriptional response to HL stress.

### Expression of Light and Heat Marker Genes in Response to High Light or Ozone Treatment

**FIGURE 2:**
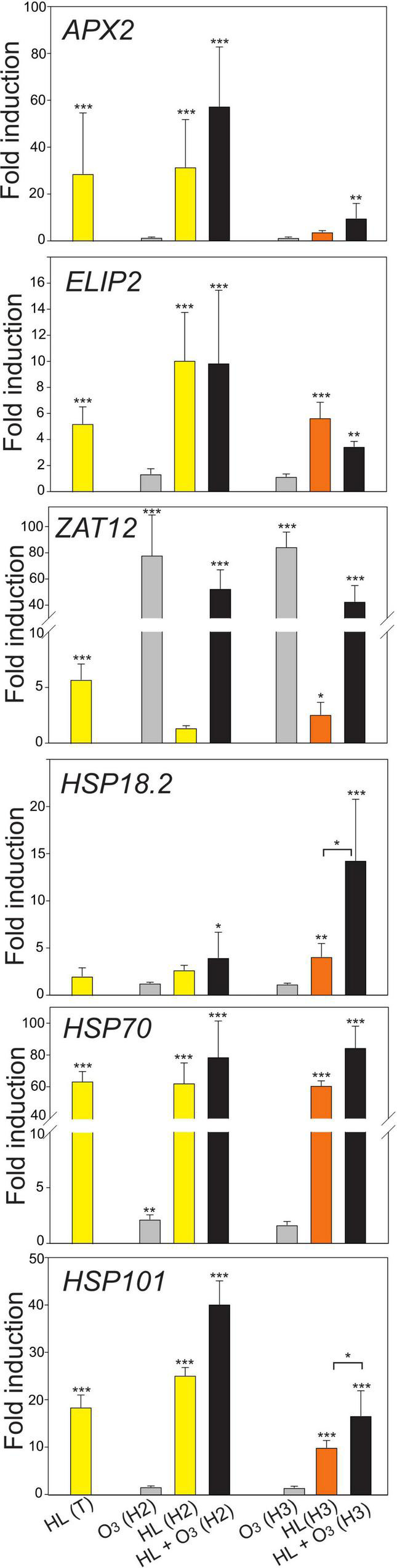
Expression of HL and heat stress marker genes, displayed as fold induction after 1 h HL, O_3_ or combined treatment. For exact experimental conditions see Table 1, T, Turku; H2, Helsinki 2; H3, Helsinki 3. The average of three biological replicates are shown; error bars depict standard deviation. Two-way ANOVA with Tukey’s test was used for statistical analysis and depicts significant differences compared to Col-0 control (*P < 0.05; **P < 0.01; ***P < 0.001).

In our experimental design, we aimed to address these questions: (1) How robust is the transcriptional response with different stress marker genes across different growth conditions? and (2) What is the difference and is there an interaction between external (apoplastic) ROS and chloroplast (HL) initiated signaling? We performed qPCR analysis using plant material obtained from different conditions, which differed with respect to the growth light intensity, photoperiod, age of the plants, and to which extent the temperature increased during the HL treatment (Table 1). While *ELIP2* transcript levels increased in all different HL treatments, *APX2* and *ZAT12* transcript levels were more variable between different growth conditions (Figure 2). *APX2* transcript levels was shown to depend on the temperature associated with the HL treatment, when the HL treatment was associated with a higher increased temperature this led to substantially increased transcript levels (Jung et al., 2013; Huang et al., 2019). In our experiments, *APX2* transcript levels was not higher when the HL treatment was associated with a +10°C increased temperature (growth condition Helsinki-3), suggesting that additional environmental factors also influence *APX2* transcript levels. In response to O_3_ (1 h, 350 nL L^–1^), transcript levels for the general ROS marker *ZAT12* increased, but there was no change in light marker genes *APX2* and *ELIP2* (Figure 2). This suggests that regulation of *APX2* and *ELIP2* transcript levels respond to signals that originate from inside the cell (chloroplast) and not from the outside (apoplast). In the combined treatment O_3_ + HL, there was no obvious interaction for these marker genes.

As the difference in growth conditions Helsinki 2 and 3 was the extent of increased temperature associated with the HL treatment, we further tested several maker genes for heat shock responses. Transcript levels for *HSP70* and *HSP101* increased in all HL treatments (Figure 2), with no obvious effect of different temperatures. After O_3_ treatment there were no or only weak transcriptional changes for the heat shock marker genes. In contrast, for the combined O_3_ + HL treatment there was increased transcript levels of *HSP18.2* and *HSP101*, especially at increased temperatures (growth condition Helsinki-3). This indicates that unlike the light stress marker gene *ELIP2*, regulation of heat shock genes respond to signals from both inside and outside the cell. Collectively our results with different light, ROS and heat shock marker genes suggests that several different marker genes should be used when testing for HL stress molecular responses. Further, the combined O_3_ + HL treatment, showed the presence of genes that are independent for combined treatment (*ELIP2*) and those that show a synergistic effect (*HSP18.2* and *HSP101*).

### The Robust High Light Molecular Response

**FIGURE 3:**
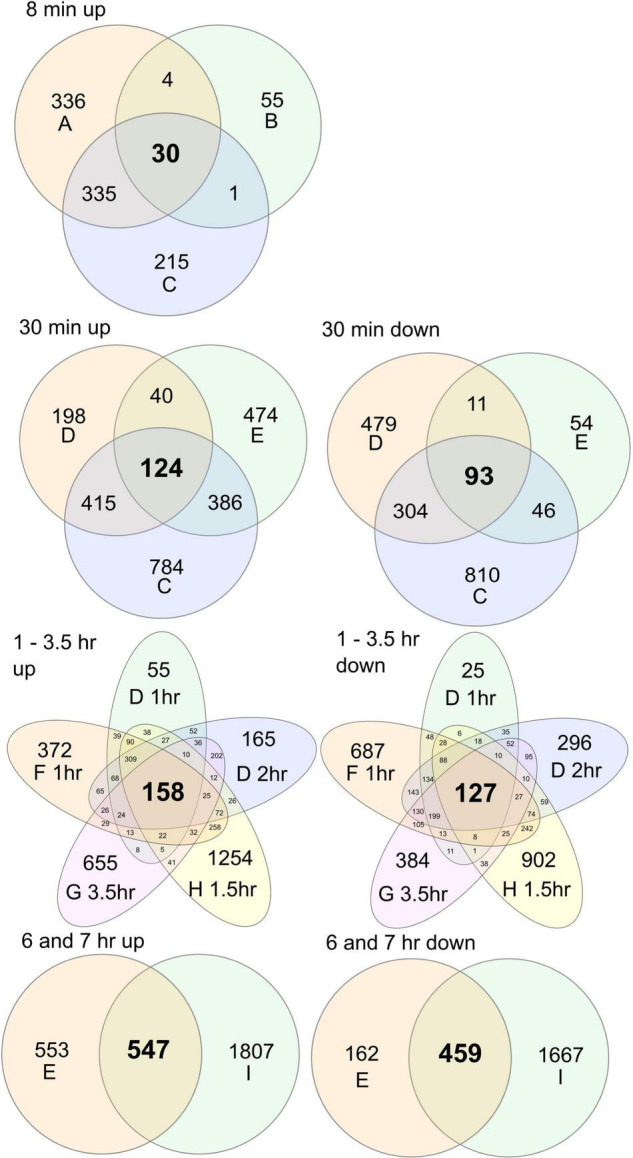
Overlap of differentially expressed genes in different high light RNA-seq datasets. Raw data was processed with the same analysis pipeline and differentially expressed genes selected at FDR corrected *P* < 0.05 and a cut-off log2 ± 1 (see Supplementary Table 2 for gene lists). For experimental summary of the datasets, see Table 1 and the original publications (referred to as A–I in figure): A, Zandalinas et al., 2019b; B, Zandalinas et al., 2020; C, Fichman et al., 2020; D, Crisp et al., 2017; E, Huang et al., 2019; F, Crisp et al., 2017; G, Alvarez-Fernandez et al., 2021; H, Zandalinas et al., 2021b; I, Balfagón et al., 2019.

To probe HL molecular responses, multiple different experimental set-ups have been used (Table 1), and as we show in our qPCR experiments, different growth conditions and the specifics of the HL treatment impact on the transcript levels for HL marker genes (Figures 1, 2). To further explore the role of differences in growth and experimental conditions (Table 1), we used all publicly available RNA-seq experiments from HL treatments (Crisp et al., 2017; Balfagón et al., 2019; Huang et al., 2019; Zandalinas et al., 2019b,^2020^, 2021b; Fichman et al., 2020; Alvarez-Fernandez et al., 2021). These experiments used different experimental designs with various mutants, time points and local versus systemic signaling; in our re-analysis we used only wild type (Col-0) samples at time points up to 7 h. In experiments with local versus systemic signaling, we used only the local treatment, i.e., the leaves that directly received HL treatment. We processed raw data through the same bioinformatics pipeline (see section “Materials and Methods”), and selected genes with FDR *P*-value < 0.05 and a twofold up or down regulation. Here it should be noted that in the publications above, some used the twofold cut-off while others did not, and since we applied this cut-off, our number of HL regulated genes were substantially less compared to the original published analysis (Supplementary Table 2). In addition, the software used to identify differentially expressed genes also has an impact on the number of differentially expressed genes found (Seyednasrollah et al., 2015; Corchete et al., 2020). As can be seen from Table 1, the RNA-seq data comes from a wide variety of growth conditions, light periods, light treatments, and plant ages. However, if it is possible to find a set of HL regulated genes that are common in these datasets, it would point toward a robust molecular response that is independent of growth conditions and plant ages. As we expected to see different genes at different time points, we focused on comparisons of similar time points (Figure 3).

At the very early time point, 8 min, there were 30 common up-regulated genes from three datasets. These included *APX2*, 10 heat shock proteins and the heat shock transcription factor *HsfA2*, previously shown to be an important regulator of HL stress responses including regulation of *APX2* transcription (Jung et al., 2013). At 8 min, there were also increased transcriptional levels for ethylene biosynthesis *ACS6* and four ethylene response factors. In the 30 min time point, there were 124 common up-regulated genes from three datasets. These included the *HsfA2* and *HsfA3* transcription factors, but surprisingly only one heat shock protein. A large number of heat shock proteins were found in the Fichman et al. (2020) (30 min) and Crisp et al. (2017) (30 min) data, but not in the Huang et al. (2019) (30 min) data, suggesting that the latter has some key difference in its experimental conditions. At 30 min, we also found the general ROS marker *ZAT12*, and the early light inducible *ELIP1*.

Three transcription factors were found in common for 30 min, which were also up-regulated at all of the other later time points (1, 1.5, 2, 3.5, 6, and 7 h): *BBX32*, *NAC13*, and *DREB2A*. As this suggest their crucial role, we will discuss them in more detail later. In the next comparison, we compared time points from 1 to 3.5 h, where we found 158 common up-regulated genes from five datasets. These included 18 heat shock proteins along with the *HsfA2* and *HsfA3* transcription factors. Finally, we compared the 6 and 7 h time points, where we found 547 common up-regulated genes. This included many enzymes from the flavonoid biosynthesis pathways including *CHS*, *CHI*, *DFR*, *F3H*, and *FLS1*, as well as the transcription factors that regulate their expression *PAP1*, *MYB11*, *MYB111*, and *TT8*. This suggest the coordinated regulation for production of protective pigments to screen HL (Ferreyra et al., 2021). Relatively few heat shock proteins were found (four) and the transcription factor *HsfA2* was not found at the late time points, indicating that transcriptional regulation of heat shock proteins is an early response to HL stress. Several regulators of JA responses (*JAZ5*, *JAZ9*, and *JAZ13*), biosynthesis of the volatile methyl jasmonate (*JASMONIC ACID CARBOXYL METHYLTRANSFERASE*) and marker gens for JA signaling (*VSP1* and *VSP2*) were up-regulated at the late time points. While we focused on the common up-regulated genes in the RNA-seq datasets, we also noted hundreds of differentially expressed genes that were unique for each dataset (Figure 3), this suggest that the plant molecular response to HL stress is largely shaped by its growth conditions.

### The Role of Abscisic Acid in Regulation of Light and Heat Stress Marker Genes

**FIGURE 4:**
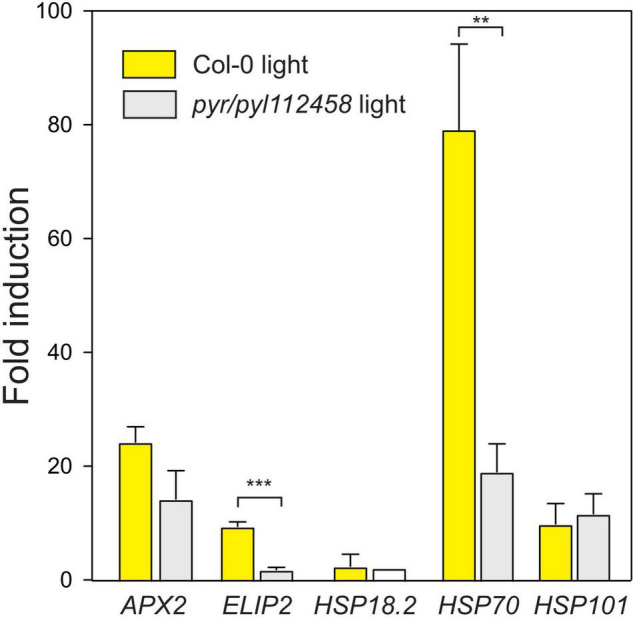
Fold induction of HL and heat stress marker genes after 1 h HL in Col-0 and *pyr/pyl112458* (growth condition Turku, Table 1). The average of three biological replicates are shown; error bars depict standard deviation. The *t*-test was used for statistical analysis and depicts significant differences between Col-0 and *pyr/pyl112458* (**P < 0.01; ***P < 0.001).

Several plant hormones, including ABA, JA, and SA are proposed regulators of HL signaling (Galvez-Valdivieso et al., 2009; Balfagón et al., 2019; Beaugelin et al., 2019; Huang et al., 2019). To evaluate the role of ABA signaling we used a strongly ABA insensitive mutant that lack six ABA receptors *pyr1 pyl1 pyl2 pyl4 pyl5 pyl8* [from here on abbreviated as *pyr/pyl112458* (Gonzalez-Guzman et al., 2012)]. In Turku growth condition (Table 1), light stress regulation of transcript levels for *ELIP2* and *HSP70* were significantly lower in *pyr/pyl112458* (Figure 4). Transcript levels for *APX2* followed the same trend, but did not reach statistical significance. In contrast, increased transcript levels for *HSP101* was independent of ABA signaling (Figure 4). We conclude that the molecular response to light stress is regulated by both ABA dependent and independent signaling pathways.

### Expression of Hormone Marker Genes in Response to High Light or Ozone Treatment

**FIGURE 5:**
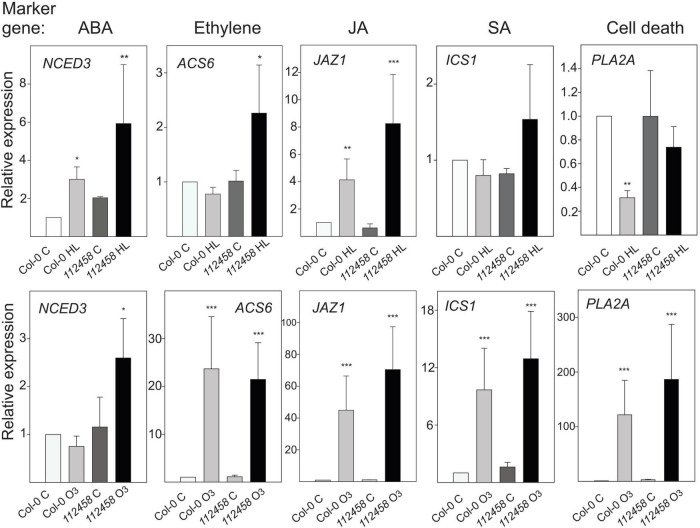
Relative expression scaled to the Col-0 control (set to 1), after 1 h HL (growth condition Turku, Table 1) or 1 h O_3_ (350 nL L^–1^, growth condition Helsinki-1) in Col-0 and *pyr/pyl112458*. The average of three biological replicates are shown; error bars depict standard deviation. Two-way ANOVA with Tukey’s test was used for statistical analysis and depicts significant differences compared to Col-0 control (*P < 0.05; **P < 0.01; ***P < 0.001).

Plant stress responses are intimately associated with several stress hormones. To follow different signaling pathways, we used marker genes related to ABA, ethylene, JA, SA, and cell death signaling (Figures 1, 5). These were tested in plants treated with 1 h HL (growth condition Turku) or 1 h O_3_ (growth condition Helsinki-1), in Col-0 and *pyr/pyl112458*. Transcript levels of the JA marker *JAZ1* increased in HL. The ABA marker gene *NCED3* was slightly elevated. However, based on *NCED3* transcript levels in several different light treatments (Figure 1), it appears that this marker gene is dependent on additional environmental factors. In contrast, the ethylene marker *ACS6* and the SA marker *ICS1* were not altered by HL and transcript levels of the cell death marker *PLA2A* significantly decreased (Figure 5).

After activation of apoplastic ROS signaling by O_3_, we observed higher transcript abundance of the ethylene marker gene *ACS6*, the SA marker gene *ICS1*, and the JA marker *JAZ1* (Figure 5). There was no apparent influence of ABA signaling on the apoplastic ROS response since *pyr/pyl1112458* displayed similar responses compared to wild type, with one exception – increased expression levels of *NCED3* was only observed by O_3_ in *pyr/pyl1112458*, possibly due to some feedback mechanism when ABA signaling is impaired. The cell death marker *PLA2A* showed the most contrasting behavior, i.e., very high transcript abundance in response to O_3_, but decreased transcript levels in response to HL.

### Identification of Genes Differentially Regulated Between Ozone and High Light

The transcriptional regulation of *PLA2A* represents an interesting case where HL and apoplastic ROS have opposite results (Figure 5). As this suggests the existence of very divergent signaling pathways, i.e., a light/chloroplast signal that leads to down-regulation and an apoplastic ROS/O_3_ signal that leads to up-regulation of the same gene, we searched for additional genes with this transcriptional profile and their biological context.

**FIGURE 6:**
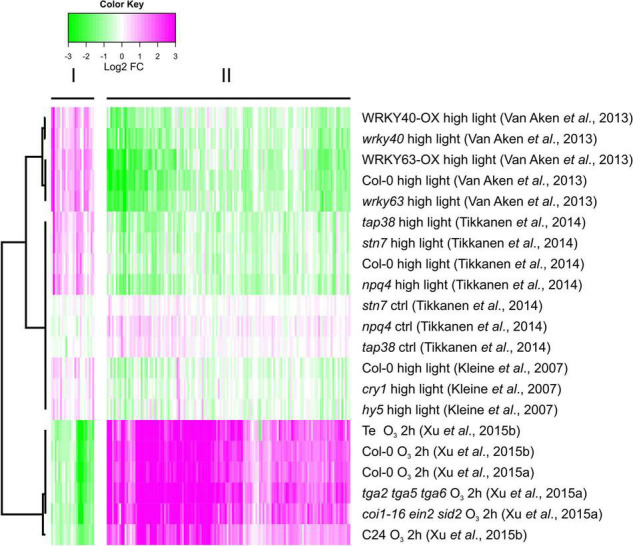
Genes with contrasting expression between HL and O_3_ – increased expression by O_3_, decreased expression by HL or vice versa, were identified from several O_3_ RNA-seq and array HL experiments. 160 genes were found (Supplementary Table 3), and subjected to bootstrapped Bayesian hierarchical clustering in log2-transformed fold changes from O_3_ RNA-seq data and HL array experiments. Magenta and green indicate increased and decreased expression compared with untreated or wild type plants, respectively.

To identify additional genes, we re-analyzed transcriptome datasets generated with arrays or RNA-seq after HL and O_3_ treatments (Kleine et al., 2007; Van Aken et al., 2013; Tikkanen et al., 2014; Xu et al., 2015a,b). We used conservative selection criteria that the genes should be significantly regulated in two independent HL experiments. We identified 160 genes with significantly altered expression (Supplementary Table 3). Of these, 136 had increased transcript levels by O_3_ and reduced transcript levels by HL; and 24 genes with increased transcript levels by HL and reduced transcript levels by O_3_. We used Bayesian hierarchical cluster analysis to analyze similarities and differences between different O_3_ and HL experiments (Figure 6). Two main clusters were identified which represents the contrasting conditions: O_3_ up, HL down and vice versa.

### The Biological Context of Genes With Contrasting Transcriptional Profiles in Response to Ozone or High Light

Next we performed GO enrichment on the genes contrasting transcriptional responses. For the 136 genes with increased transcript levels by O_3_ and decreased transcript levels after HL there was enrichment of multiple biological processes related to stress, pathogen responses and cell death (Supplementary Table 4). In the GO category molecular function, kinase, and signal transduction were enriched. In the list of 24 genes with decreased transcript levels by O_3_ and increased transcript levels by HL, there was an enrichment of biological processes related to regulation of metabolic processes, transcription and gene expression; and in molecular function there was an enrichment for transcription factors (Supplementary Table 4).

**FIGURE 7:**
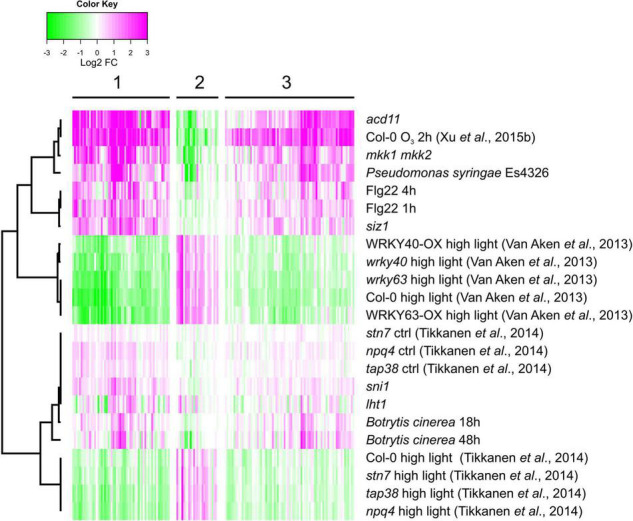
Pathogen regulation of genes with contrasting expression between HL and O_3_. Gene expression data sets include treatments with pathogens or flg22. In addition, mutants undergoing spontaneous cell death were included (see section “Materials and Methods” for full list of experiments). Genes were subjected to bootstrapped Bayesian hierarchical clustering of log2-transformed fold changes. Magenta and green indicate increased and decreased expression compared with untreated or wild type plants, respectively.

Reactive oxygen species acts as signaling molecules in defense against pathogens and in regulation of cell death. We analyzed expression levels in transcriptome datasets from several pathogen infections and lesion mimic mutants that undergo spontaneous cell death (Figure 7). For comparison, two of the HL datasets and one of the O_3_ samples were included. Interestingly, a majority of the genes that had increased transcript levels by O_3_ (and decreased transcript levels by HL) also had increased transcript levels in response to *P. syringae* infection, flg22 treatment and in the mutants *acd11* and *mkk1 mkk2* that undergo spontaneous cell death. Thus, both GO enrichment and the expression profile of genes with contrasting O_3_ versus HL transcriptional profiles, indicated that they have a role in defense responses, particularly related to pathogen infection and cell death. As the HL regulation of these genes were opposite to O_3_, pathogen and cell death, this suggests the possibility of a signal from the chloroplast that could interact with other ROS signaling pathways.

### Confirmation of Genes With Contrasting Ozone and High Light Transcriptional Responses

The HL datasets used in the cluster analysis contained a variety of different time points and growth conditions (Table 1). To further support the results of the cluster analysis we selected several genes, four with increased transcript levels by O_3_ and decreased transcript levels by HL and one gene with decreased transcript level by O_3_ and increased transcript level by HL. These were tested in qPCR at 1 h time point in Col-0 and *pyr/pyl112458*. All five genes behaved as expected in the O_3_ experiment (Supplementary Figure 2). In HL the four genes with decreased expression levels were reproduced (*At1g33590*, *At3g02410*, *At3g47800*, and *MLO2*). For the last gene with increased expression level in HL (*FATTY ALCOHOL OXIDASE 3* – *FAO3*), it had higher transcript abundance which did not reach statistical significance (Supplementary Figure 2). We included these genes in our analysis of datasets from Genevestigator (Figure 1). Also, in this analysis the selected genes behaved as expected, *At1g33590*, *At3g02410*, *At3g47800*, and *MLO2* had decreased transcript levels and *FAO3* increased transcript levels in multiple different HL datasets (Figure 1). We observed that *At1g33590*, *At3g02410*, and *At3g47800* had decreased transcript levels in multiple datasets from ABA treatments (Figure 1). Results from the *pyr/pyl112458* mutant suggested that HL down regulation of *At1g33590* and *MLO2* could require functional ABA signaling (Supplementary Figure 2).

The main focus of the meta-analysis of gene expression data was to identify genes with opposite regulation by O_3_ versus HL. However, we also identified genes with similar regulation of transcript abundance (Supplementary Table 5). Among genes with increased transcript levels by both treatments were JA biosynthesis and signaling genes (*AOC3*, *LOX4*, *JAZ1*, and *JAZ6*) and ROS response genes (*ZAT10* and *ZAT12*). The expression of these genes was consistent with the qPCR results (Figures 2, 5). Numerous GO categories associated with abiotic stress, ROS signaling and HL responses but not cell death were associated with genes with increased expression levels by both O_3_ and HL (Supplementary Table 5).

### Combined Ozone and High Light Treatments

**FIGURE 8:**
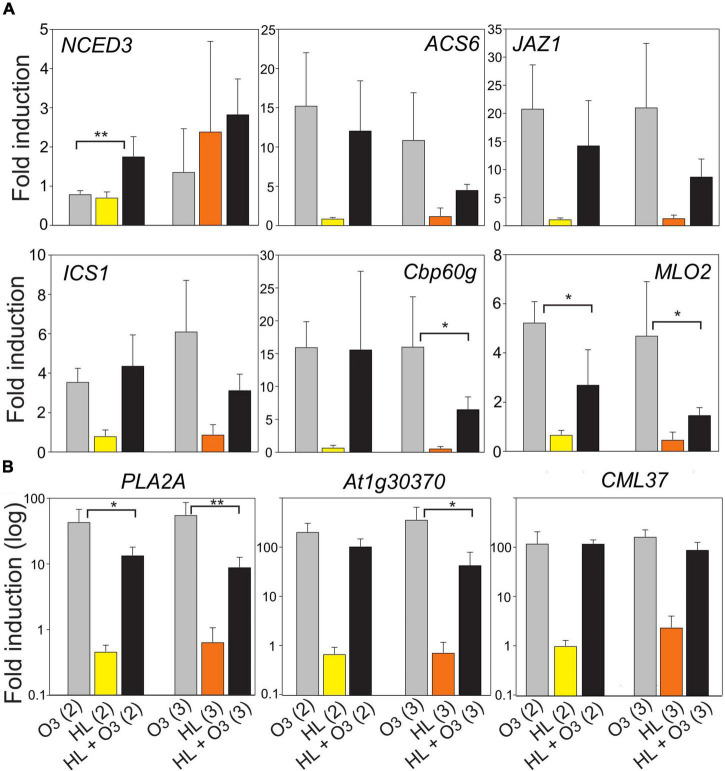
Fold induction of selected marker genes after 1 h HL, 1 h O_3_ (350 nL L^– 1^) or combined treatment (growth conditions Helsinki-2 and Helsinki-3, Table 1). Panel **(A)** has normal scale and panel **(B)** log10 scale, to better visualize the large differences in transcript levels between samples. The average of four biological replicates are shown; error bars depict standard deviation. The *t*-test was used for statistical analysis and depicts significant differences between O_3_ and combined O_3_ + HL (*P < 0.05; **P < 0.01).

To directly test the interaction between apoplastic ROS (O_3_) and chloroplast signals (HL), we used HL treatments inside our O_3_ chambers (growth conditions Helsinki-2 and 3, Table 1 and Figures 2, 8). For the heat stress markers genes, but not light stress marker genes, we observed synergistic effects between HL, O_3_, and increased temperature (Figure 2). We expanded this analysis to the hormone marker genes to get further information on potential interaction between the apoplast and chloroplast signaling pathways. Based on the GO analysis and cluster analysis (Figure 7), we also included three genes with increased transcript abundance early after pathogen treatment *At1g30370*, *CML37*, and *Cbp60g* (Jacob et al., 2018). In multiple datasets from Genevestigator, *At1g30370* and *Cbp60g* [which encodes a transcription factor that regulate expression of the SA biosynthesis gene *ICS1* (Wang et al., 2011)], had increased transcript levels by biotic stress and O_3_ and decreased transcript levels by HL (Figure 1). In contrast, *CML37* had increased transcript levels by both HL and O_3_.

In the combined treatments, there appeared to be a stronger effect when there was also an additional increase in temperature (growth condition Helsinki-3) for the marker genes *Cbp60g, MLO2*, *PLA2A*, and *At1g30370*, in which the combined treatment led to significant reduction in transcript levels compared to O_3_ alone (Figure 8). This significant reduction in combined treatment was also observed for growth condition Helsinki-2 and the marker genes *MLO2* and *PLA2A* (Figure 8), where the heat component was minimal (Table 1). In addition, there was a trend toward lower transcript levels in combined treatments for *ACS6* and *JAZ1*, although this did not reach statistical significance. We conclude that a signal pathway initiated from the chloroplast (HL treatment) can down regulate transcript levels for pathogen related genes as a single treatment (Figures 1, 7 and Supplementary Figure 2), but also in the combined treatment where this chloroplast signal can modulate and partially block the signaling initiated from the apoplast (O_3_) (Figure 8).

### Regulation of Cell Death After Combined Ozone + High Light

**FIGURE 9:**
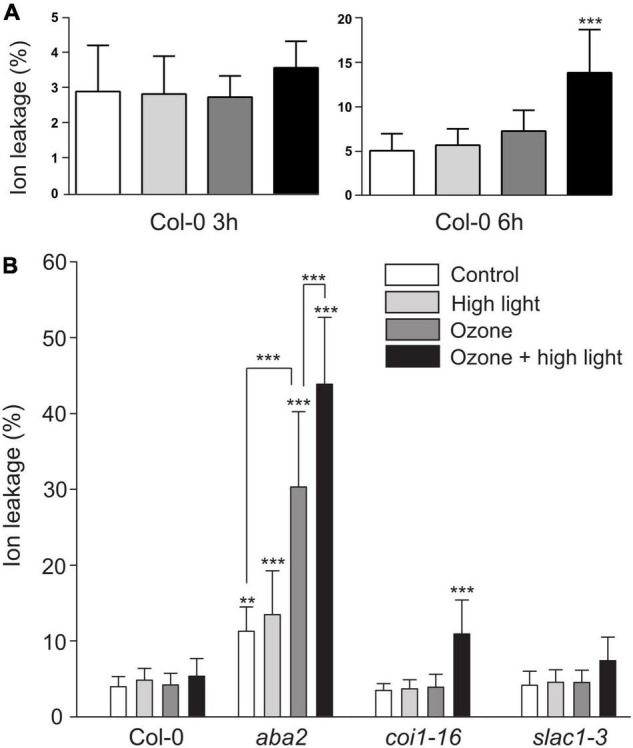
Cell death after combined O_3_ and HL. **(A)** Cell death quantified with ion leakage after 3 or 6 h of HL, O_3_ (350 nL L^– 1^) or combined treatment (growth condition Helsinki-3). **(B)** Plants treated with 2 h of HL, O_3_ or combined treatment followed by 4 h in control conditions and quantification of cell death. The average of three biological replicates are shown (*N* = 15); error bars depict standard deviation. One-way or two-way ANOVA with Tukey’s test was used for statistical analysis and depicts significant differences compared to Col-0 control (*P < 0.05; **P < 0.01; ***P < 0.001).

A characteristic response to O_3_ in sensitive plants is accumulation of ROS leading to induction of cell death (Wohlgemuth et al., 2002). To test the relevance of the interaction between O_3_ and HL, and if HL modulates O_3_ cell death, we measured cell death in Col-0 at 3 and 6 h after the combined treatment (Figure 9A, growth condition Helsinki-3). We quantified cell death as ion leakage and observed an increase in cell death only at 6 h in the combined treatment.

Jasmonic acid is a regulator of O_3_ cell death (Xu et al., 2015a), and ABA a regulator of HL cell death. To test the role of these hormones in the O_3_ + HL interaction we used *aba2*, a strong ABA biosynthesis mutant and *coi1-16*, a mutant for the JA receptor. As an additional control, we included the O_3_ sensitive *slac1* as this mutant is O_3_ sensitive independent of hormone signaling due to more open stomata that allow high O_3_ uptake (Vahisalu et al., 2008). In a first trial, the combined treatment severely damaged all three mutants, preventing precise quantification of cell death. Therefore, we used a shorter 2 h treatment plus recovery at 4 h in control conditions followed by quantification of cell death. In this scheme with a lower O_3_ dose, mutants that previously were shown to be O_3_ sensitive [*slac1*, *coi1* (Vahisalu et al., 2008; Xu et al., 2015a)], did not show damage by O_3_ alone (Figure 9B). In contrast, *aba2* showed higher ion leakage already in control conditions, possibly due to the permeable cuticle of ABA deficient mutants (Cui et al., 2016). The *aba2* mutant was also highly O_3_ sensitive, which might be related to the very high stomatal conductance of this mutant (Merilo et al., 2018). In the combined treatment, HL + raised temperature increased the damaging effects of O_3_ in *aba2* and *coi1* (Figure 9). Thus, a combined stress treatment cause more damage than O_3_ alone.

## Discussion

### The Role of Reactive Oxygen Species in Plant Signal Networks

Signaling pathways in plants are highly responsive to ROS produced in different cellular compartments, but the mechanisms underlying appropriate ROS-induced responses upon biotic and abiotic challenges are only starting to emerge (Castro et al., 2021). The ROS signaling network is integrated with the stress hormone signaling network: in O_3_ (apoplastic ROS), SA and ethylene promotes cell death and JA is protective (Xu et al., 2015a); in HL induced cell death, JA and SA promotes cell death (Laloi and Havaux, 2015; Beaugelin et al., 2019). However, assigning specific and clear-cut roles for hormones is often an over simplification, since growth conditions, genetic background, or stress severity can change the extent of cell death. For example, SA can also protect against O_3_ (Xu et al., 2015b) and HL induced cell death (Lv et al., 2015). Most likely, the balance between different signaling pathways determines the outcome of a stress treatment.

As plants in nature are likely to experience multiple altered growth conditions simultaneously, using experimental set-ups where two stress conditions interact can further help to understand the priority and interaction between different signaling pathways. Meta-analysis of array and RNA-seq data from different stress combinations show that all stress combinations tested so far result in transcriptome changes that are unique for each specific stress combination (Zandalinas et al., 2019a). This indicates that plants are highly responsive to changes in the environment with corresponding activation of signaling pathways that integrate multiple sources of signals. Site specific ROS production could be one of the ways that plants use to activate different signaling pathways; demonstrated by *methylene blue sensitivity* mutants, which show impaired HL regulation of ^1^O_2_ regulated genes but not H_2_O_2_/O_2_.^–^ regulated genes (Shao et al., 2013).

This study further illustrates that signals from different subcellular compartments have different signaling roles, since O_3_ and HL had very different outcomes on expression of several marker genes (Figures 1, 5). In particular, the cell death marker *PLA2A* showed opposite regulation – increased transcript levels by O_3_ and decreased transcript levels by HL. Additional genes with contrasting expression profiles in O_3_ versus HL were identified from transcriptome datasets and were enriched for pathogen response genes (Figure 7). To directly evaluate the interaction between apoplast and chloroplast signaling, we used combined O_3_ + HL treatments. While O_3_ had no effect on HL marker genes, there was a consistent inhibitory effect of HL on O_3_ regulated genes associated with cell death and pathogen responses (Figure 8). This emphasizes that: (1) apoplastic and chloroplastic ROS activate distinct signaling pathways; (2) at least one signal initiated from HL converge with the apoplastic ROS signal to regulate changes in transcript levels for genes related to pathogen infection and cell death.

### The Impact of Different Growth Conditions on Plant Stress Responses

A challenge in interpretation of results from plant stress studies is the variety of different growth conditions used in research with Arabidopsis. In one of the few studies that directly attempted to replicate similar growth conditions in ten different laboratories, revealed significant changes in growth and metabolite profiles due to subtle variations in growth conditions (Massonnet et al., 2010). For HL experiments, considerably different growth conditions and HL treatments have been used (Table 1), which in turn is likely to give difference in molecular responses measured as altered transcript levels (Figure 3). These experiments used a wide range of plant ages and different light periods (constant light, 16:8, 12:12, or 8:16 light/dark). Another critical factor in HL experiments is the potential increase in temperature associated with the HL treatment. For example, the HL marker gene *APX2* show very high transcript levels when the HL treatment is combined with increased temperature (Jung et al., 2013; Huang et al., 2019). Even small increases in temperature are monitored by plants through PhyB and PIF4 (PHYTOCHROME INTERACTING FACTOR 4), and PIF4 acts a negative regulator of transcript levels for pathogen defense related genes (Legris et al., 2016; Gangappa et al., 2017). Analysis of HL RNA-seq data from contrasting growth conditions (Table 1 and Figure 3), gives further support for both a robust response to HL and growth condition specific HL responses. We suggest that a robust molecular response to HL stress require a core set of transcription factors to execute the transcriptional regulation. We found consistent up-regulation of *HsfA2* (and to lesser extent *HsfA3*) in all data-sets for early time points from 8 min to 3.5 h; and up-regulation of *NAC13*, *DREB2A*, and *BBX32* in all data-sets from 30 min to 7 h. This is consistent with the proposed role for HsfA2/HsfA3 as key positive regulators for early HL responses (Jung et al., 2013); and the newly established role for BBX32 as a key negative regulator of HL responses (Alvarez-Fernandez et al., 2021). BBX32 was proposed to act to down-regulate pathogen defense related genes after HL exposure (Alvarez-Fernandez et al., 2021), consistent with our cluster analysis which identified genes up-regulated by O_3_ treatments, but down-regulated by HL treatment (Figures 1, 7). NAC13 has not previously been associated with HL stress, but this transcription factors is a key regulator of mitochondrial retrograde signaling (De Clercq et al., 2013; Shapiguzov et al., 2019). Its consistent up-regulation across all time points from 30 min to 7 h, suggest that the HL stress response require coordinated responses from both the chloroplast and mitochondria. DREB2A has been extensively characterized for its role in drought stress responses, and it also acts as a regulator of heat stress responses (Sakuma et al., 2006). Accordingly, the consistent up-regulation of *DREB2A*, *HsfA2*, and *HsfA3* across many time points, suggest their coordinated function to regulate the expression of heat shock proteins.

As changes in the light environment may be one of the most common experiences by plants in nature, it is perhaps not surprising that transcriptional responses to HL is integrated with growth conditions. HL stress has been studied in combination with other stresses including heat (Balfagón et al., 2019), drought (Giraud et al., 2008), and heat, salt and chloroplast ROS generated from methyl viologen (Zandalinas et al., 2021b); where the combined treatments increased the amount of damage compared to single treatments. Higher concentration of O_3_ in the troposphere is significantly correlated with both increase in temperature and sunshine hours (Tiwari et al., 2008). The impact of O_3_ pollution on yield of agriculturally important species, including wheat and rice, is higher in field experiments than in pot experiments, pointing toward interactions between O_3_ and other unknown cues from the environment (Feng et al., 2022). Further identification of signals from the environment that modulate O_3_ responses is crucial to protect against yield losses from O_3_ pollution, which can be as high as 30% in wheat (Feng et al., 2022). Here we showed that O_3_ + HL led to more damage than O_3_ alone in Arabidopsis (Figure 9), this observation makes it possible to use this model plant to better understand how O_3_ interacts with other environmental factors including light (Juran et al., 2021).

Retrograde signaling from the chloroplast involve multiple signaling molecules (Leister, 2019). ABA is critical for plant drought and cold responses, and is an important signaling molecule in response to HL based on several evidences. HL treatment led to increased expression of ABA biosynthesis enzymes and production of ABA (Galvez-Valdivieso et al., 2009). The ABA biosynthesis double mutant *nced3 nced5* showed increased damage after HL treatment (Huang et al., 2019), and HL induction of *APX2* and *ELIP2* was impaired in ABA biosynthesis and signaling mutants (Fryer et al., 2003; Galvez-Valdivieso et al., 2009; Figure 4). However, the role of ABA in HL signaling also appears to be cell specific and influenced by environmental factors (Gorecka et al., 2014). In contrast, for the marker genes tested here (Figure 5), ABA does not appear to regulate O_3_ transcriptional responses.

Plants constantly face different signals from the environment that needs to be integrated with developmental programs. Here we have shown that HL activates signaling that can inhibit signaling initiated from the apoplast, which could be used by the plant to prioritize between potentially conflicting defense responses.

## Data Availability Statement

The datasets presented in this study can be found in online repositories. The names of the repositories and accession numbers can be found in the Materials and Methods, and in Supplementary Table 2.

## Author Contributions

MB conceived and designed the experiments and wrote the manuscript. EX, MT, and MB performed the experiments. EX, MT, FS, SK, and MB analyzed the data. All authors read and approved the final manuscript.

## Conflict of Interest

The authors declare that the research was conducted in the absence of any commercial or financial relationships that could be construed as a potential conflict of interest.

## Publisher’s Note

All claims expressed in this article are solely those of the authors and do not necessarily represent those of their affiliated organizations, or those of the publisher, the editors and the reviewers. Any product that may be evaluated in this article, or claim that may be made by its manufacturer, is not guaranteed or endorsed by the publisher.

## Footnotes

1
www.valoya.com

2
https://www.qbaseplus.com/

3
http://arabidopsis.info/affy/link_to_iplant.html

4
http://www.ncbi.nlm.nih.gov/geo

5
http://www.ebi.ac.uk/arrayexpress/

## Supplementary Material

Supplementary Figure 1 Genes co-expressed with *APX2*, *ELIP2*, and *ZAT12* were identified with the Arabidopsis Coexpression Tool and displayed in a cladogram (Zogopoulos et al., 2021).

Supplementary Figure 2 Relative expression scaled to the Col-0 control (set to 1), after 1 h HL (growth condition Turku, Table 1) or 1 h O_3_ (350 nL L^–1^, growth condition Helsinki-1) in Col-0 and *pyr/pyl112458*. The average of three biological replicates are shown; error bars depict standard deviation. Two-way ANOVA with Tukey’s test was used for statistical analysis and depicts significant differences compared to Col-0 control (**P* < 0.05; ^**^*P* < 0.01; ^***^*P* < 0.001).

Supplementary Table 1 Primers used in this study.

Supplementary Table 2 Differentially expressed genes in HL RNA-seq datasets and the overlap of differentially expressed genes, corresponding to the Venn diagram analysis in Figure 3.

Supplementary Table 3 Genes with increased expression by O_3_ and decreased expression by HL (or vice versa), corresponding to the cluster analysis in Figures 6, 7.

Supplementary Table 4 Gene Ontology (GO) analysis of genes with increased expression by O_3_ and decreased expression by HL (or vice versa).

Supplementary Table 5 Genes with increased expression by O_3_ and HL, or decreased expression by O_3_ and HL.

## References

[B1] Alvarez-Fernandez R. Penfold C. A. Galvez-Valdivieso G. Exposito-Rodriguez M. Stallard E. J. Bowden L. (2021). Time-series transcriptomics reveals a BBX32-directed control of acclimation to high light in mature *Arabidopsis* leaves. *Plant J.* 107 1363–1386. 10.1111/tpj.15384 34160110

[B2] Balfagón D. Sengupta S. Gómez-Cadenas A. Fritschi F. B. Azad R. Mittler R. (2019). Jasmonic acid is required for plant acclimation to a combination of high light and heat stress. *Plant Physiol.* 181 1668–1682. 10.1104/pp.19.00956 31594842 PMC6878009

[B3] Balfagon D. Zandalinas S. I. Mittler R. Gomez-Cadenas A. (2020). High temperatures modify plant responses to abiotic stress conditions. *Physiol. Plant.* 170 335–344. 10.1111/ppl.13151 32533896

[B4] Beaugelin I. Chevalier A. D’Alessandro S. Ksas B. Novak O. Strnad M. (2019). OXI1 and DAD regulate light-induced cell death antagonistically through jasmonate and salicylate levels. *Plant Physiol.* 180 1691–1708. 10.1104/pp.19.00353 31123095 PMC6752932

[B5] Bechtold U. Richard O. Zamboni A. Gapper C. Geisler M. Pogson B. (2008). Impact of chloroplastic- and extracellular-sourced ROS on high light-responsive gene expression in Arabidopsis. *J. Exp. Bot*. 59, 121–133. 10.1093/jxb/erm289 18212028

[B6] Castro B. Citterico M. Kimura S. Stevens D. M. Wrzaczek M. Coaker G. (2021). Stress-induced reactive oxygen species compartmentalization, perception and signalling. *Nat. Plants* 7 403–412. 10.1038/s41477-021-00887-0 33846592 PMC8751180

[B7] Corchete L. A. Rojas E. A. Alonso-Lopez D. De Las Rivas J. Gutierrez N. C. Burguillo F. J. (2020). Systematic comparison and assessment of RNA-seq procedures for gene expression quantitative analysis. *Sci. Rep.* 10:19737. 10.1038/s41598-020-76881-x 33184454 PMC7665074

[B8] Crisp P. A. Ganguly D. R. Smith A. B. Murray K. D. Estavillo G. M. Searle I. (2017). Rapid recovery gene downregulation during excess-light stress and recovery in *Arabidopsis*. *Plant Cell* 29 1836–1863. 10.1105/tpc.16.00828 28705956 PMC5590493

[B9] Cui F. Q. Brosche M. Lehtonen M. T. Amiryousefi A. Xu E. J. Punkkinen M. (2016). Dissecting abscisic acid signaling pathways involved in cuticle formation. *Mol. Plant.* 9 926–938. 10.1016/j.molp.2016.04.001 27060495

[B10] De Clercq I. Vermeirssen V. Van Aken O. Vandepoele K. Murcha M. W. Law S. R. (2013). The membrane-bound NAC transcription factor ANAC013 functions in mitochondrial retrograde regulation of the oxidative stress response in *Arabidopsis*. *Plant Cell* 25 3472–3490. 10.1105/tpc.113.117168 24045019 PMC3809544

[B11] Du Z. Zhou X. Ling Y. Zhang Z. H. Su Z. (2010). agriGO: a GO analysis toolkit for the agricultural community. *Nucleic Acids Res.* 38 W64–W70. 10.1093/nar/gkq310 20435677 PMC2896167

[B12] Feng Z. Z. Xu Y. S. Kobayashi K. Dai L. L. Zhang T. Y. Agathokleous E. (2022). Ozone pollution threatens the production of major staple crops in East Asia. *Nat. Food* 3 47–56. 10.1038/s43016-021-00422-6 37118490

[B13] Ferreyra M. L. F. Serra P. Casati P. (2021). Recent advances on the roles of flavonoids as plant protective molecules after UV and high light exposure. *Physiol. Plant.* 173 736–749. 10.1111/ppl.13543 34453749

[B14] Fichman Y. Zandalinas S. I. Sengupta S. Burks D. Myers R. J. Azad R. K. (2020). MYB30 orchestrates systemic reactive oxygen signaling and plant acclimation. *Plant Physiol.* 184 666–675. 10.1104/pp.20.00859 32699028 PMC7536697

[B15] Frenkel M. Kulheim C. Jankanpaaa H. J. Skogstrom O. Dall’Osto L. Agren J. (2009). Improper excess light energy dissipation in *Arabidopsis* results in a metabolic reprogramming. *BMC Plant Biol.* 9:12. 10.1186/1471-2229-9-12 19171025 PMC2656510

[B16] Fryer M. J. Ball L. Oxborough K. Karpinski S. Mullineaux P. M. Baker N. R. (2003). Control of ascorbate peroxidase 2 expression by hydrogen peroxide and leaf water status during excess light stress reveals a functional organisation of *Arabidopsis* leaves. *Plant J.* 33 691–705. 10.1046/j.1365-313X.2003.01656.x 12609042

[B17] Gadjev I. Vanderauwera S. Gechev T. S. Laloi C. Minkov I. N. Shulaev V. (2006). Transcriptomic footprints disclose specificity of reactive oxygen species signaling in *Arabidopsis*. *Plant Physiol.* 141 436–445. 10.1104/pp.106.078717 16603662 PMC1475436

[B18] Galvez-Valdivieso G. Fryer M. J. Lawson T. Slattery K. Truman W. Smirnoff N. (2009). The high light response in *Arabidopsis* involves ABA signaling between vascular and bundle sheath cells. *Plant Cell* 21 2143–2162. 10.1105/tpc.108.061507 19638476 PMC2729609

[B19] Gangappa S. N. Berriri S. Kumar S. V. (2017). PIF4 coordinates thermosensory growth and immunity in *Arabidopsis*. *Curr. Biol.* 27 243–249. 10.1016/j.cub.2016.11.012 28041792 PMC5266789

[B20] Gautier L. Cope L. Bolstad B. M. Irizarry R. A. (2004). affy - analysis of Affymetrix GeneChip data at the probe level. *Bioinformatics* 20 307–315. 10.1093/bioinformatics/btg405 14960456

[B21] Giraud E. Ho L. H. M. Clifton R. Carroll A. Estavillo G. Tan Y. F. (2008). The absence of alternative oxidase1a in *Arabidopsis* results in acute sensitivity to combined light and drought stress. *Plant Physiol.* 147 595–610. 10.1104/pp.107.115121 18424626 PMC2409015

[B22] Gollan P. J. Tikkanen M. Aro E. M. (2015). Photosynthetic light reactions: integral to chloroplast retrograde signalling. *Curr. Opin. Plant Biol.* 27 180–191. 10.1016/j.pbi.2015.07.006 26318477

[B23] Gonzalez-Guzman M. Pizzio G. A. Antoni R. Vera-Sirera F. Merilo E. Bassel G. W. (2012). *Arabidopsis* PYR/PYL/RCAR receptors play a major role in quantitative regulation of stomatal aperture and transcriptional response to abscisic acid. *Plant Cell* 24 2483–2496. 10.1105/tpc.112.098574 22739828 PMC3406898

[B24] Gorecka M. Alvarez-Fernandez R. Slattery K. McAusland L. Davey P. A. Karpinski S. (2014). Abscisic acid signalling determines susceptibility of bundle sheath cells to photoinhibition in high light-exposed *Arabidopsis* leaves. *Philos. Trans. R. Soc. B. Biol. Sci.* 369:20130234. 10.1098/rstb.2013.0234 24591719 PMC3949397

[B25] Hayami N. Sakai Y. Kimura M. Saito T. Tokizawa M. Iuchi S. (2015). The responses of *Arabidopsis* early light-induced protein2 to ultraviolet B, high light, and cold stress are regulated by a transcriptional regulatory unit composed of two elements. *Plant Physiol.* 169 840–855. 10.1104/pp.15.00398 26175515 PMC4577391

[B26] Heberle H. Meirelles G. V. da Silva F. R. Telles G. P. Minghim R. (2015). InteractiVenn: a web-based tool for the analysis of sets through venn diagrams. *BMC Bioinformatics* 16:169. 10.1186/s12859-015-0611-3 25994840 PMC4455604

[B27] Hellemans J. Mortier G. De Paepe A. Speleman F. Vandesompele J. (2007). qBase relative quantification framework and software for management and automated analysis of real-time quantitative PCR data. *Genome Biol.* 8:R19. 10.1186/gb-2007-8-2-r19 17291332 PMC1852402

[B28] Hruz T. Laule O. Szabo G. Wessendorp F. Bleuler S. Oertle L. (2008). Genevestigator v3: a reference expression database for the meta-analysis of transcriptomes. *Adv. Bioinform.* 2008:420747. 10.1155/2008/420747 19956698 PMC2777001

[B29] Huang J. Zhao X. Chory J. (2019). The *Arabidopsis* transcriptome responds specifically and dynamically to high light stress. *Cell Rep.* 29 4186–4199.e. 10.1016/j.celrep.2019.11.051 31851942 PMC7030938

[B30] Jacob F. Kracher B. Mine A. Seyfferth C. Blanvillain-Baufume S. Parker J. E. (2018). A dominant-interfering camta3 mutation compromises primary transcriptional outputs mediated by both cell surface and intracellular immune receptors in *Arabidopsis thaliana*. *New Phytol.* 217 1667–1680. 10.1111/nph.14943 29226970 PMC5873390

[B31] Jung H. S. Crisp P. A. Estavillo G. M. Cole B. Hong F. X. Mockler T. C. (2013). Subset of heat-shock transcription factors required for the early response of *Arabidopsis* to excess light. *Proc. Natl. Acad. Sci. U.S.A.* 110 14474–14479. 10.1073/pnas.1311632110 23918368 PMC3761602

[B32] Juran S. Grace J. Urban O. (2021). Temporal changes in ozone concentrations and their impact on vegetation. *Atmosphere* 12:82. 10.3390/atmos12010082

[B33] Kallio M. A. Tuimala J. T. Hupponen T. Klemela P. Gentile M. Scheinin I. (2011). Chipster: user-friendly analysis software for microarray and other high-throughput data. *BMC Genomics* 12:507. 10.1186/1471-2164-12-507 21999641 PMC3215701

[B34] Karpinski S. Reynolds H. Karpinska B. Wingsle G. Creissen G. Mullineaux P. (1999). Systemic signaling and acclimation in response to excess excitation energy in *Arabidopsis*. *Science* 284 654–657. 10.1126/science.284.5414.654 10213690

[B35] Kleine T. Kindgren P. Benedict C. Hendrickson L. Strand A. (2007). Genome-wide gene expression analysis reveals a critical role for CRYPTOCHROME1 in the response of arabidopsis to high irradiance. *Plant Physiol.* 144 1391–1406. 10.1104/pp.107.098293 17478635 PMC1914119

[B36] Krasensky-Wrzaczek J. Kangasjarvi J. (2018). The role of reactive oxygen species in the integration of temperature and light signals. *J. Exp. Bot.* 69 3347–3358. 10.1093/jxb/ery074 29514325

[B37] Laloi C. Havaux M. (2015). Key players of singlet oxygen-induced cell death in plants. *Front. Plant Sci.* 6:39. 10.3389/fpls.2015.00039 25699067 PMC4316694

[B38] Legris M. Klose C. Burgie E. S. Rojas C. C. Neme M. Hiltbrunner A. (2016). Phytochrome B integrates light and temperature signals in *Arabidopsis*. *Science* 354 897–900. 10.1126/science.aaf5656 27789798

[B39] Leister D. (2019). Piecing the puzzle together: the central role of reactive oxygen species and redox hubs in chloroplast retrograde signaling. *Antioxid. Redox Signal.* 30 1206–1219. 10.1089/ars.2017.7392 29092621

[B40] Lim S. D. Kim S. H. Gilroy S. Cushman J. C. Choi W. G. (2019). Quantitative ROS bioreporters: a robust toolkit for studying biological roles of ROS in response to abiotic and biotic stresses. *Physiol. Plant.* 165 356–368. 10.1111/ppl.12866 30411793

[B41] Littlejohn G. R. Breen S. Smirnoff N. Grant M. (2021). Chloroplast immunity illuminated. *New Phytol.* 229 3088–3107. 10.1111/nph.17076 33206379

[B42] Lv F. F. Zhou J. Zeng L. Z. Xing D. (2015). beta-cyclocitral upregulates salicylic acid signalling to enhance excess light acclimation in *Arabidopsis*. *J. Exp. Bot.* 66 4719–4732. 10.1093/jxb/erv231 25998906

[B43] Massonnet C. Vile D. Fabre J. Hannah M. A. Caldana C. Lisec J. (2010). Probing the reproducibility of leaf growth and molecular phenotypes: a comparison of three arabidopsis accessions cultivated in ten laboratories. *Plant Physiol.* 152 2142–2157. 10.1104/pp.109.148338 20200072 PMC2850010

[B44] Merilo E. Yarmolinsky D. Jalakas P. Parik H. Tulva I. Rasulov B. (2018). Stomatal VPD response: there is more to the story than ABA. *Plant Physiol.* 176 851–864. 10.1104/pp.17.00912 28986421 PMC5761775

[B45] Michelet L. Krieger-Liszkay A. (2012). Reactive oxygen intermediates produced by photosynthetic electron transport are enhanced in short-day grown plants. *Biochim. Biophys. Acta* 1817 1306–1313. 10.1016/j.bbabio.2011.11.014 22172734

[B46] Palma K. Thorgrimsen S. Malinovsky F. G. Fiil B. K. Nielsen H. B. Brodersen P. (2010). Autoimmunity in *Arabidopsis* acd11 is mediated by epigenetic regulation of an immune receptor. *PLoS Pathog.* 6:e1001137. 10.1371/journal.ppat.1001137 20949080 PMC2951382

[B47] Phua S. Y. De Smet B. Remacle C. Chan K. X. Van Breusegem F. (2021). Reactive oxygen species and organellar signaling. *J. Exp. Bot.* 72 5807–5824. 10.1093/jxb/erab218 34009340

[B48] Putri G. H. Anders S. Pyl P. T. Pimanda J. E. Zanini F. (2022). Analysing high-throughput sequencing data in Python with HTSeq 2.0. *Bioinformatics* 38, 2943–2945. 10.1093/bioinformatics/btac166 35561197 PMC9113351

[B49] Ritchie M. E. Phipson B. Wu D. Hu Y. F. Law C. W. Shi W. (2015). limma powers differential expression analyses for RNA-sequencing and microarray studies. *Nucleic Acids Res.* 43:e47. 10.1093/nar/gkv007 25605792 PMC4402510

[B50] Robinson M. D. McCarthy D. J. Smyth G. K. (2010). edgeR: a Bioconductor package for differential expression analysis of digital gene expression data. *Bioinformatics* 26 139–140. 10.1093/bioinformatics/btp616 19910308 PMC2796818

[B51] Sakuma Y. Maruyama K. Qin F. Osakabe Y. Shinozaki K. Yamaguchi-Shinozaki K. (2006). Dual function of an *Arabidopsis* transcription factor DREB2A in water-stress-responsive and heat-stress-responsive gene expression. *Proc. Natl. Acad. Sci. U.S.A.* 103 18822–18827. 10.1073/pnas.0605639103 17030801 PMC1693746

[B52] Seyednasrollah F. Laiho A. Elo L. L. (2015). Comparison of software packages for detecting differential expression in RNA-seq studies. *Brief. Bioinform.* 16 59–70. 10.1093/bib/bbt086 24300110 PMC4293378

[B53] Shao N. Duan G. Y. Bock R. (2013). A mediator of singlet oxygen responses in *Chlamydomonas reinhardtii* and *Arabidopsis* identified by a luciferase-based genetic screen in algal cells. *Plant Cell* 25 4209–4226. 10.1105/tpc.113.117390 24151292 PMC3877789

[B54] Shapiguzov A. Vainonen J. P. Hunter K. Tossavainen H. Tiwari A. Jarvi S. (2019). *Arabidopsis* RCD1 coordinates chloroplast and mitochondrial functions through interaction with ANAC transcription factors. *Elife* 8:e43284. 10.7554/eLife.43284 30767893 PMC6414205

[B55] Simkova K. Moreau F. Pawlak P. Vriet C. Baruah A. Alexandre C. (2012). Integration of stress-related and reactive oxygen species-mediated signals by Topoisomerase VI in *Arabidopsis thaliana*. *Proc. Natl. Acad. Sci. U.S.A.* 109 16360–16365. 10.1073/pnas.1202041109 22988090 PMC3479551

[B56] Tikkanen M. Aro E. M. (2014). Integrative regulatory network of plant thylakoid energy transduction. *Trends in Plant Sci.* 19 10–17. 10.1016/j.tplants.2013.09.003 24120261

[B57] Tikkanen M. Gollan P. J. Mekala N. R. Isojarvi J. Aro E. M. (2014). Light-harvesting mutants show differential gene expression upon shift to high light as a consequence of photosynthetic redox and reactive oxygen species metabolism. *Philos. Trans. R. Soc. B. Biol. Sci.* 369:20130229. 10.1098/rstb.2013.0229 24591716 PMC3949394

[B58] Tiwari S. Rai R. Agrawal M. (2008). Annual and seasonal variations in tropospheric ozone concentrations around Varanasi. *Int. J. Remote Sens.* 29 4499–4514. 10.1080/01431160801961391

[B59] Vaahtera L. Brosche M. Wrzaczek M. Kangasjarvi J. (2014). Specificity in ROS signaling and transcript signatures. *Antioxid. Redox Signal.* 21 1422–1441. 10.1089/ars.2013.5662 24180661 PMC4158988

[B60] Vahisalu T. Kollist H. Wang Y. F. Nishimura N. Chan W. Y. Valerio G. (2008). SLAC1 is required for plant guard cell S-type anion channel function in stomatal signalling. *Nature* 452 487–U415. 10.1038/nature06608 18305484 PMC2858982

[B61] Vainonen J. P. Kangasjarvi J. (2015). Plant signalling in acute ozone exposure. *Plant Cell Environ.* 38 240–252. 10.1111/pce.12273 24417414

[B62] Van Aken O. Zhang B. T. Law S. Narsai R. Whelan J. (2013). AtWRKY40 and AtWRKY63 modulate the expression of stress-responsive nuclear genes encoding mitochondrial and chloroplast proteins. *Plant Physiol.* 162 254–271. 10.1104/pp.113.215996 23509177 PMC3641207

[B63] Wang L. Tsuda K. Truman W. Sato M. Nguyen L. V. Katagiri F. (2011). CBP60g and SARD1 play partially redundant critical roles in salicylic acid signaling. *Plant J.* 67 1029–1041. 10.1111/j.1365-313X.2011.04655.x 21615571

[B64] Waszczak C. Carmody M. Kangasjarvi J. (2018). Reactive oxygen species in plant signaling. *Ann. Rev. Plant Biol.* 69 209–236. 10.1146/annurev-arplant-042817-040322 29489394

[B65] Wohlgemuth H. Mittelstrass K. Kschieschan S. Bender J. Weigel H. J. Overmyer K. (2002). Activation of an oxidative burst is a general feature of sensitive plants exposed to the air pollutant ozone. *Plant Cell Environ.* 25 717–726. 10.1046/j.1365-3040.2002.00859.x

[B66] Wrzaczek M. Brosche M. Salojarvi J. Kangasjarvi S. Idanheimo N. Mersmann S. (2010). Transcriptional regulation of the CRK/DUF26 group of receptor-like protein kinases by ozone and plant hormones in *Arabidopsis*. *BMC Plant Biol.* 10:95. 10.1186/1471-2229-10-95 20500828 PMC3095361

[B67] Xu E. J. Vaahtera L. Brosche M. (2015a). Roles of defense hormones in the regulation of ozone-induced changes in gene expression and cell death. *Mol. Plant* 8 1776–1794. 10.1016/j.molp.2015.08.008 26348016

[B68] Xu E. J. Vaahtera L. Horak H. Hincha D. K. Heyer A. G. Brosche M. (2015b). Quantitative trait loci mapping and transcriptome analysis reveal candidate genes regulating the response to ozone in *Arabidopsis thaliana*. *Plant Cell Environ.* 38 1418–1433. 10.1111/pce.12499 25496229

[B69] Zabala M. D. T. Littlejohn G. Jayaraman S. Studholme D. Bailey T. Lawson T. (2015). Chloroplasts play a central role in plant defence and are targeted by pathogen effectors. *Nat. Plants* 1:15074. 10.1038/nplants.2015.74 27250009

[B70] Zandalinas S. I. Fichman Y. Devireddy A. R. Sengupta S. Azad R. K. Mittler R. (2020). Systemic signaling during abiotic stress combination in plants. *Proc. Natl. Acad. Sci. U.S.A.* 117 13810–13820. 10.1073/pnas.2005077117 32471943 PMC7306788

[B71] Zandalinas S. I. Fritschi F. B. Mittler R. (2019a). Signal transduction networks during stress combination. *J. Exp. Bot.* 71, 1734–1741. 10.1093/jxb/erz486 31665392

[B72] Zandalinas S. I. Fritschi F. B. Mittler R. (2021a). Global warming, climate change, and environmental pollution: recipe for a multifactorial stress combination disaster. *Trends Plant Sci.* 26 588–599. 10.1016/j.tplants.2021.02.011 33745784

[B73] Zandalinas S. I. Sengupta S. Burks D. Azad R. K. Mittler R. (2019b). Identification and characterization of a core set of ROS wave-associated transcripts involved in the systemic acquired acclimation response of *Arabidopsis* to excess light. *Plant J.* 98 126–141. 10.1111/tpj.14205 30556340 PMC6850305

[B74] Zandalinas S. I. Sengupta S. Fritschi F. B. Azad R. K. Nechushtai R. Mittler R. (2021b). The impact of multifactorial stress combination on plant growth and survival. *New Phytol.* 230 1034–1048. 10.1111/nph.17232 33496342 PMC8048544

[B75] Zhang S. R. Apel K. Kim C. H. (2014). Singlet oxygen-mediated and EXECUTER-dependent signalling and acclimation of *Arabidopsis thaliana* exposed to light stress. *Philos. Trans. R. Soc. B. Biol. Sci.* 369:20130227. 10.1098/rstb.2013.0227 24591714 PMC3949392

[B76] Zogopoulos V. L. Saxami G. Malatras A. Angelopoulou A. Jen C.-H. Duddy W. J. (2021). *Arabidopsis* coexpression tool: a tool for gene coexpression analysis in *Arabidopsis thaliana*. *iScience* 24:102848. 10.1016/j.isci.2021.102848 34381973 PMC8334378

