# Supplementary figures and images for: Simultaneous Ozone and High Light Treatments Reveal an Important Role for the Chloroplast in Co-ordination of Defense Signaling
Source: Front Plant Sci. 2022 Jul 7;13:883002. doi: 10.3389/fpls.2022.883002 (PMC9303991; doi:10.3389/fpls.2022.883002)

Supplementary Figure 1

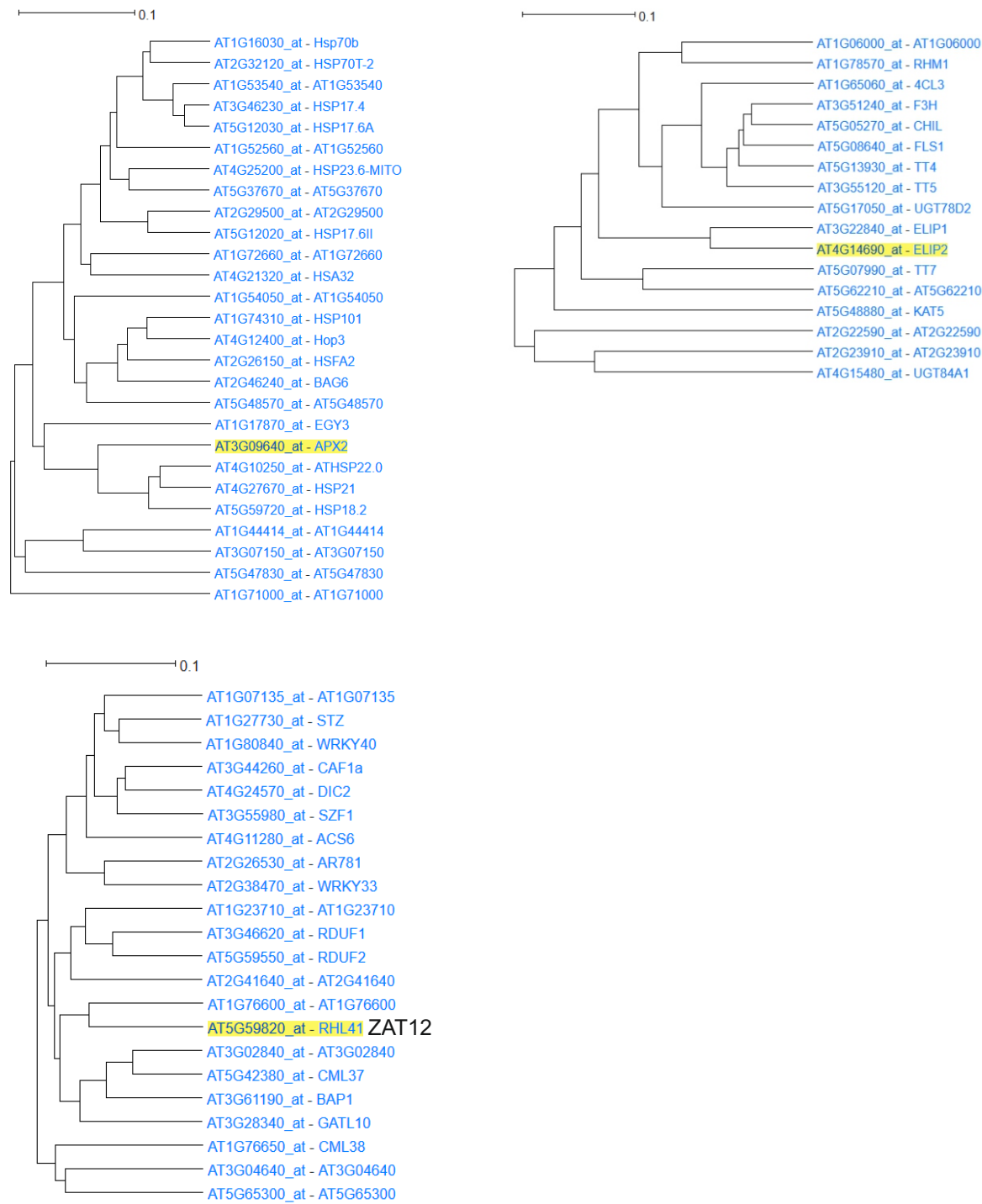

Supplement: Supplementary Figure 1 — Genes co-expressed with APX2, ELIP2, and ZAT12 were identified with the Arabidopsis Coexpression Tool and displayed in a cladogram (Zogopoulos et al., 2021). [file Data_Sheet_1.PDF]

## Supplementary Figure 2

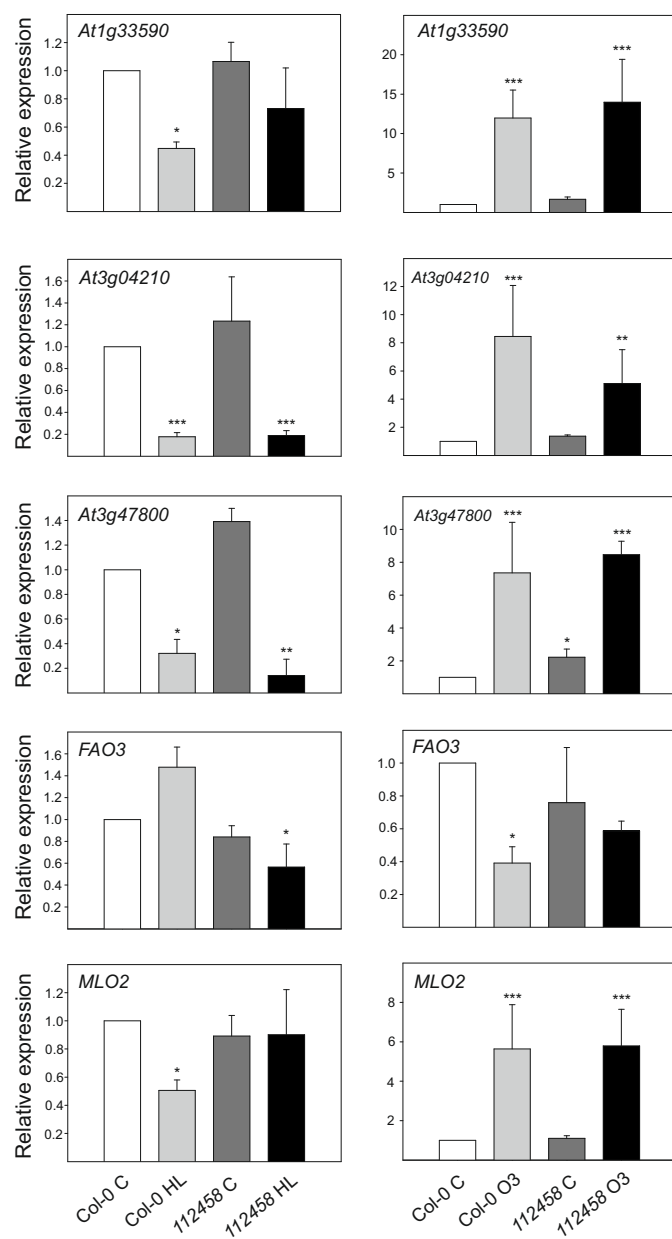

Supplement: Supplementary Figure 2 — Relative expression scaled to the Col-0 control (set to 1), after 1 h HL (growth condition Turku, Table 1) or 1 h O3 (350 nL L–1, growth condition Helsinki-1) in Col-0 and pyr/pyl112458. The average of three biological replicates are shown; error bars depict standard deviation. Two-way ANOVA with Tukey’s test was used for statistical analysis and depicts significant differences compared to Col-0 control (*P < 0.05; **P < 0.01; ***P < 0.001). [file Data_Sheet_2.PDF]
